# Does a four-week intensive scoliosis-specific exercises programme improve body-image in subjects with idiopathic scoliosis and is the effect rated equally by patients, physiotherapists and an external rater with scoliosis?

**DOI:** 10.1186/1748-7161-7-S1-O52

**Published:** 2012-01-27

**Authors:** J Head, E Maude, J Black, T Rolfe, R Dorman

**Affiliations:** 1 Scoliosis SOS Clinic, Martlesham, UK

## Background

Improving trunk appearance is important for scoliotic patients. Selecting an appropriate rater is vital for effectively measuring this outcome. This study investigates whether a four-week intensive scoliosis-specific exercise programme results in improved patient body image and how this varies between patients, physiotherapists and an external scoliotic rater.

## Materials and methods

82 patients (70 females 12 males) with IS and mean age 30.79 years (Range 10-81) were treated with a four-week intensive scoliosis-specific physiotherapy course (ScolioGold). Patients, 2 blinded physiotherapists and a blinded scoliotic rater rated patients’ body-image before and after treatment. Body-image was assessed using a 0-10 scale, for 5 elements (Head, Shoulders, Ribs, Waist and Hips).

## Results

Mean total scores before treatment were; patients 28.51 (SD8.76), physiotherapists 22.94 (SD6.01) and external rater 20.55 (SD7.27) and after treatment were; patients 15.46 (SD7.40), physiotherapists 13.73 (SD5.88) and external rater 6.61 (SD4.10). Differences in patients (P), physiotherapists (T) and external rater(E) body-image scores were found to have statistically significantly improved after treatment using Wilcoxon-signed rank (p =<0.001, p=<0.001, p=<0.001). ICC scores between P&T, P&E and T&E were; fair (0.28), slight (0.19) and moderate (0.58) before treatment and fair (0.34), fair (0.28), moderate (0.59) after treatment.

## Conclusions

Statistically significant improvements between pre-post treatment scores substantiate intensive exercise methods (ScolioGold), in the treatment of IS-related negative body image. Significant variation between patients’, physiotherapists’ and external rater’s scoring highlight the need for patients to rate body-image due to the subjectivity of this outcome measure, to ensure we adopt a client-centred care approach in our treatment goals to improve patient body-image.

